# Which family physician should I choose? The analytic hierarchy process approach for ranking of criteria in the selection of a family physician

**DOI:** 10.1186/s12911-015-0183-1

**Published:** 2015-08-05

**Authors:** Emel Kuruoglu, Dilek Guldal, Vildan Mevsim, Tolga Gunvar

**Affiliations:** 1 Department of Statistics/Computer Science, Dokuz Eylul University Faculty of Science, Izmir, Turkey; 2 Department of Family Medicine, Dokuz Eylul University Faculty of Medicine, Dokuz Eylül Universitesi Tıp Fakültesi Aile Hekimliği, AD 35340 İnciraltı İzmir, Turkey

**Keywords:** Analytic hierarchy process, Criteria for the choosing family physician, Patient

## Abstract

**Background:**

Choosing the most appropriate family physician (FP) for the individual, plays a fundamental role in primary care. The aim of this study is to determine the selection criteria for the patients in choosing their family doctors and priority ranking of these criteria by using the multi-criteria decision-making method of the Analytic Hierarchy Process (AHP) model.

**Methods:**

The study was planned and conducted in two phases. In the first phase, factors affecting the patients’ decisions were revealed with a qualitative research. In the next phase, the priorities of FP selection criteria were determined by using AHP model. Criteria were compared in pairs. 96 patient were asked to fill the information forms which contains comparison scores in the Family Health Centres.

**Results:**

According to the analysis of focus group discussions FP selection criteria were congregated in to five groups: Individual Characteristics, Patient-Doctor relationship, Professional characteristics, the Setting, and Ethical Characteristics.

For each of the 96 participants, comparison matrixes were formed based on the scores of their information forms. Of these, models of only 5 (5.2 %) of the participants were consistent, in other words, they have been able to score consistent ranking. The consistency ratios (CR) were found to be smaller than 0.10. Therefore the comparison matrix of this new model, which was formed based on the medians of scores only given by these 5 participants, was consistent (CR = 0.06 < 0.10).

According to comparison results; with a 0.467 value-weight, the most important criterion for choosing a family physician is his/her ‘Professional characteristics’.

**Conclusions:**

Selection criteria for choosing a FP were put in a priority order by using AHP model. These criteria can be used as measures for selecting alternative FPs in further researches.

## Background

Decision making doesn’t necessarily mean reaching a right or wrong conclusion. Decision making means choosing one of the available options. What is important in decision-making is not starting from the beginning and aiming to find what is correct, but reaching a decision based upon the final analyses after all the different alternatives have been compared. Another distinguishing factor between decisions and reality is the fact that decisions have a subjective quality. In other words, choices can differ not only from person to person, but they can be changed by the individual himself at any given moment.

Even though medical sciences are considered as a branch of positive sciences, they are still fundamentally different due to the fact that they encompass many subjective processes. Contrary to mathematics and chemistry that are based on definite concepts, they incorporate possibilities and decisions based on choices depending on the situation. Within this framework, it is clear that the health services offer a series of choices for both the providers and the receivers. The decision of which service is to be provided to whom is planned according to the shifting balance of various factors that vary over time, for example prevalence of illnesses, funds allocated for health, and the prioritized need groups. On the other hand, it is also affected by many other various factors such as the public’s experience of health services and their attitudes towards illness, their beliefs concerning health, and their overall expectations. Even when choosing their physicians, people are influenced by many different factors. Determining these factors and identifying their impact on the decision-making process will not only aid the availability of health services but also will help in the planning of efforts to create awareness in both the physicians and the public.

A good patient-doctor relationship and continuity of care in healthcare are two of the main principles guiding family physicians which have a significant effect on health outcomes [1–6]. Choosing the most appropriate physician for the individual plays a fundamental role in both establishing and maintaining a continuous and effective patient-doctor relationship.

There are many studies investigating the criteria used by patients when choosing their doctors [7–10]. These studies have gained more importance based on the acceptance of the patient centred care approach. Studies have shown that factors such as being examined by the physician himself, previous acquaintance, not having to wait too long, flexibility in appointment hours, sincerity, provision of clear and abundant information, follow-ups, extensive consultations, and shared decision making play a role in the choice of physician [5, 9–12].

However priorities and importance of these factors may differ for every individual as well as may vary for the same individual in different times. In order to meet their expectations, patients should reflect their priorities accurately to their decision making processes for choosing a physician. But the human brain can only put few options in a priority order simultaneously. This leads to failure in reflection of individual’s own priorities to selection process, especially when there are a lot of selection criteria. For accurate reflection of the expectations proper weighing of the priorities is necessary.

There have been many quantitative and qualitative studies in order to reveal the factors that influence patients’ preferences in choosing a physician. Some quantitative researches, such as surveys often used in cross sectional studies, weighting distributions, randomized controlled trials with regression analysis, discrete choice experiments, and structural equation models have also been implemented [9, 10, 13, 14]. These studies were mostly trying to describe patients’ decision making but not help them to give a tool they need. We think that the AHP model will provide a useful tool for patients to reflect their expectations in their selections.

Although the AHP is used for the analysis of the decision-making process [15–17] in the studies for the clinical decision-making [18–22], there is not much literature about concerning patients’ choices. Many factors are involved in the patients’ preferences of physician. These changes may result from factors and priorities that are available. Inconsistencies in the decision affect patient satisfaction and prevent the development of a good patient-doctor relationship. In this situation, if the decision is not coincide with expectations and preferences, then there can be serious problems.

The aim of this study is to determine the selection criteria for the patients in choosing their family doctors and priority ranking of these criteria by using the AHP model.

## Methods

The study was conducted as part of a project to evaluate the implementation of the “Analytic Hierarchy Process” used by the public when choosing family physicians. [23]. The ethical approval was given by the Dokuz Eylul University Ethics Committee for Non-Interventional Studies. Written informed consent for participation in the study was obtained from participants.

The study was planned and conducted in two phases. The first was conducted in four family health care centres from different socioeconomically levels in Izmir. Qualitative research design was used in the first phase. Participants were selected by typical case sampling method and focus group interview was chosen as the data collection method.

Typical case sampling is a type of purposeful sampling used in qualitative research in which, “subjects are selected who are likely to behave as most of their counterparts would”. [24] This purposeful sampling technique is used for investigating phenomenon generally seen in the universe. When choosing the samples, we consulted with the physicians in family health care centres because they know much about their registered population.

For selecting the focus group participants, the physicians were asked to recommend the names of those patients registered to them whom they treated on a daily basis, according to health problems, reasons for visiting the health care centre, the frequency of visits, and relationships with the health care workers and volunteer.

**Focus group** interview is a form of qualitative research data collecting method in which a group of people are asked about their perceptions, opinions, beliefs, and attitudes towards a product, service, concept, advertisement, idea, or packaging. Questions are asked in an interactive group setting where participants are free to talk with other group members.

Focus group interview were completed in three sessions with a total of 30 participants. Some of the characteristics of participants were shown in Table 1.

**Table 1:** The demographic characteristics of participants of focus groups

Characteristics	Frequency
Gender	
Man	12
Woman	18
Age	
18–24	2
25–34	5
35–44	9
45–54	8
55 and above	6
Educational Level	
Primary school and below	18
Secondary school	9
University	3

Focus group interviews were facilitated by two of the authors who were experienced in this field. During the interviews, participants were asked about their views on important features they were looking for when they are choosing their family physician and how their family doctors should be. All interviews were audio-taped. Later, these records were decoded into text and analyzed by three researchers. Themes were determined. The grouping of criteria was conducted independently by the researchers who then discussed and agreed on the main criteria.

The results of the qualitative research conducted in the first phase were published in a paper entitled “Do the core competencies of family medicine relevant to patients’ expectations?” [25].

In the second phase, patients who visiting the family health care facilities between 01.03.2010 and 17.03.2010, evaluate the criteria by using the forms. Participants were consisted of 96 individuals who were older than 18 years old, admitted to the family health centres due to any health problem and were volunteered to participate in the study. Data were collected with the face to face interviews. Due to the characteristics of AHP method, for reliability of the results, probability sampling was not necessary. The first part of the form consists of questions about socio-demographic characteristics where as second part consists of items for comparison of criteria in the form of 0 – 9 scale. For each of these forms AHP model was applied and criteria for each individual evaluated in Expert Choice (EC) software. Evaluation of 5 out of 96 participants was found to be consistent. New AHP model was formed and criteria were put in a priority order based on the medians of these five individuals.

### The analytic hierarchy process

The Analytic Hierarchy Process (AHP) is a multi-criterion decision-making technique created by Thomas L. Saaty [1977]. Compared to other approaches, the main distinguishing feature of the AHP is the direct consideration and application of personal judgment.

AHP algorithm is basically composed of two steps:

1. Determine the relative weights of the decision criteria.
2. Determine the relative rankings (priorities) of alternatives. In some studies, only first step can be used to rank criteria.

*First of all, a* hierarchy is determined defining the problem. The purpose is placed at the top. The criteria are placed below this main level. Finally, if second step will be applied, alternatives are placed at the bottom.

AHP primarily based on one to one comparisons related with a decision hierarchy which use a predefined comparison scale either with factors affecting the decisions or with the importance of these factors on decision points.

**Fig. 1:**
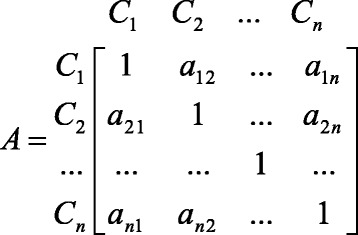
Matrix of paired comparisons

A is the comparison matrix of size n × n, for n criteria, also called the priority matrix (Fig. 1) [16].

Where

$$ \mathrm{n} = \mathrm{t}\mathrm{he}\ \mathrm{number}\ \mathrm{o}\mathrm{f}\ \mathrm{criteria}\ \mathrm{t}\mathrm{o}\ \mathrm{be}\ \mathrm{evaluated}, $$

$$ {\mathrm{C}}_{\mathrm{i}} = \mathrm{i}\mathrm{t}\mathrm{h}\ \mathrm{criterion}, $$

$$ {\mathrm{a}}_{\mathrm{ij}} = \mathrm{t}\mathrm{he}\ \mathrm{i}\mathrm{mportance}\ \mathrm{o}\mathrm{f}\ \mathrm{criterion}\ \mathrm{i}\ \mathrm{t}\mathrm{o}\ \mathrm{criterion}\ \mathrm{j}\ \left({\mathrm{a}}_{\mathrm{ji}} = 1/{\mathrm{a}}_{\mathrm{ij}}\mathrm{and}\ {\mathrm{a}}_{\mathrm{ii}} = 1\right). $$

If criterion C_i_ is more important as a_ij_ than criterion C_j,;_ then criterion C_j_ is 1/a_ij_ times more important than criterion C_i_. The preferences are expressed as 1 to 9 point scale provided in Table 2. Intermediate values not provided in the table may be used to form a comparison matrix. It is assumed that the alternatives are independent when expressing preferences in AHP model. Comparison matrix is formed to determine weight of priority without considering alternatives.

**Table 2:** The paired comparisons scale for AHP

Intensity of importance	Definitions
9	Extreme importance
7	Very strong importance
5	Strong importance
3	Moderate importance
1	Equal importance (preference)
2,4,6,8	Intermediate values

AHP evaluations are based on the assumption that the decision maker is rational, i.e., if A is preferred to B and B is preferred to C, then A is preferred to C.

Although AHP is a consistent model within itself, the factualness of the results inherently depend upon the consistency of the one to one comparisons of the factors by the decision maker. AHP offers a process which can measure the consistency of these comparisons. The calculation of Consistency Ratio (CR) gives us a priority vector(W) and also provides an opportunity for one to one comparison of factors. If CR value is smaller than 0.10 it means that the comparisons are consistent. CR value greater than 0.10 means that either a calculation mistake in the model or inconsistency of the comparisons of decision maker. Using the Expert Choice program, it is possible to determine the priorities and consistency ratios [26].

## Results

### The characteristics of the participants of second phase

The average age of the participants was 38,1 ± 15,9, of which 33.3 % were males and 66.7 % were females. 69.8 % were married, 51 % were high school graduates or above, 74 % were parents, and 55.2 % were living in households with 4–5 people. 56.3 % were in a middle class income bracket and 85.4 % had social security. The characteristics of the participants are shown in Table 3.

**Table 3:** The Demographic characteristics of the participants

Characteristics	Category	n	Percent (%)
Gender	Female	64	66,7
	Male	32	33,3
Age	Under 30	31	32,3
	30-50	43	44,8
	Over 50	22	22,9
Marital Status	Married/living together	67	69,8
	Living alone	29	30,2
Education	Middle school [max]	47	49,0
	High School and above	49	51,0
Economic Status	Good	26	27,1
	Medium	54	56,3
	Poor	16	16,7
Social Security	Yes	82	85,4
	No	14	14,6
Number of Household members	1-3	33	34,4
	4-5	53	55,2
	6 and more	10	10,4
Number of Children	None	25	26,0
	1	21	21,9
	2	32	33,3
	3 or more	18	18,8

Percent 25 of the participants [n = 24] received care from the health centres on a continuous basis, while 69.8 % [n = 67] received occasional care and 5.2 % [n = 5] indicated that they had never used the centres. 31.3 % [n = 30] of the applicants suffered from chronic diseases while 68.7 % [n = 66] did not. 62.5 % [n = 60] indicated that upon becoming ill, they had applied directly to the centres as a primary care while 37.5 % [n = 36] had applied as a secondary and tertiary care

### Focus group interviews and the criteria

Criteria have been classified as five main criteria groups according to the evaluation of focus group interviews: **Individual Characteristics, Patient-Doctor relationship, Professional characteristics, the Setting,** and **Ethical Characteristics.** The characteristics classified according to these main criteria groups are provided in Table 4.

**Table 4:** Criteria for Choosing a Family Physician

Individual Characteristics	Patient-Doctor relationship	Professional Characteristics	The Setting	Ethical Characteristics
Smiling, Gentle, Polite, Respectful, Understanding, Non-frightening, Calm and not nervous, Friendly	Closeness, Makes a patient feel valued, Recognises a patient, Provides medicine without charge, Listens and hears, Solves payment problems in some manner, Wants to see you again, Informative, Knows your district, Explains in laymen’s’ terms, Sincere, Listens in depth, One of the family, Knows our family and children, We can talk to him, We can laugh with him, The doctor should know me, I should know the doctor	Gets a medical history, Knowledgably, Experienced, Ability to diagnose and treat without referrals elsewhere, Authority to write all prescriptions, Ability to write medical reports to enable the patient to get medication, Trustworthy [someone who won’t misdiagnose], Ability to rewrite the same medications, Doesn’t rush but pays attention [does not worry about malpractice], Asks questions [about the issue], Examines, Understands us, Can consult other doctors, Can cooperate with other doctors, Can understand our ailments	Not crowded, Don’t have to wait, Good organisation [queuing, numbering, door signs etc.], Should have only one doctor, If s/he changes, then everything starts all over again, Should honour the queue	In emergency situations, shouldn’t consider who is responsible for the patient, Should have ethical values [the service he provides shouldn’t be commensurate with the salary he receives], Should write a medical report when needed, Should not distinguish between the health clinic and private practice, Should focus on his job and shouldn’t be distracted by other things [such as chatting with medical firm representatives], Should not discriminate according to ethnic origin, Should not discriminate according to social security availability, Should be trustworthy [in terms of his relationship with medical firms],

### AHP application

The form prepared to determine the prioritization of the characteristics incorporated when choosing a family physician was administered to 96 patients and an AHP model was created.

**Fig. 2:**
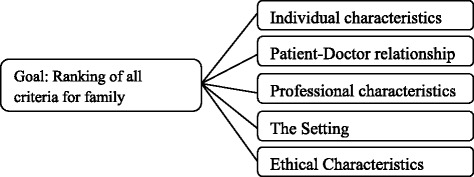
The AHP model incorporating the purpose and criteria

The hierarchy model incorporating the objectives and criteria when choosing a physician can be seen in Fig. 2.

In the form completed by 96 patients who visited the family health care services, the responses concerning the prioritisation of the criteria were calculated according to the Expert Choice program and the consistency ratios of the paired comparisons were analysed. In 91 of the 96 patients [94.8 %], the preference based on priorities was greater than 0.10; the consistency ratios were found to be less than 0.10 for 5[5.2 %] patients. 94.8 % of patients were inconsistent in ranking importance of the features they are looking for in an physician. Their minds are not clear about which criteria is more important for them. Only 5 % of the participants could rank the importance of these features consistently. So, the order of priority derived only from comparison matrix of this 5 %.

The consistency ratios that were less than 0.10 for the 5 patient’s information forms were then analysed; a median was calculated according to the importance ratings and a new model was prepared. The paired comparisons of the numerical values of the criteria are given in Table 5 for the new model.

**Table 5:** Paired comparisons of the prioritising of the criteria when choosing family physicians

	Individual characteristics	Patient-Doctor relationship	Professional characteristics	The setting	Ethical characteristics
Individual characteristics	1	1/3	1/5	3	2
Patient-Doctor relationship	3	1	1/3	4	2
Professional Characteristics	5	3	1	5	3
The Setting	1/3	1/4	1/5	1	1
Ethical Characteristics	1/2	1/2	1/3	1	1

Consistency Ratio = 0.06

For example, the “Patient-Doctor Relationship” criterion is slightly more important (3), and therefore more preferred, than the “Individual Characteristics” one in the prioritisation of the criteria when choosing a family physician. In the numerical comparisons, it can be observed that the “Professional Characteristics” criterion with a value of 5, has more importance than the “The Setting” criterion. All the criteria were compared with one another in this way and the prioritization table illustrated in Table 5 was determined.

In comparison Table 5, it is clear that, with an consistency ratio of 0.06 which is less than 0.1, the model works.

**Fig. 3:**
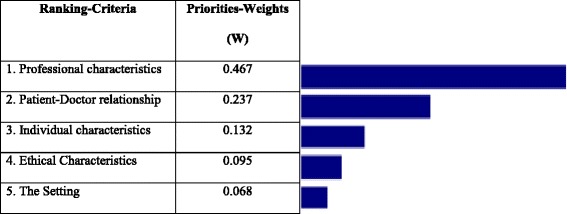
Ranking of all criteria for FP selection

Figure 3 clearly illustrates the fact that, with a 0.467 value, the most important criterion when choosing a family physician is his/her Professional Characteristics. Accordingly, it can be said that patients consider the doctor’s professional characteristics to be the most important point in determining their choice of physician. The other criteria are Patient-Doctor Relationship, Individual Characteristics, Ethical Characteristics and the Setting respectively.

## Discussion

In this study, it is clear that ‘Professional Characteristics’ of FP were most important criteria in patients’ choices. Within this scope are factors such as the ability of the physician to familiarise himself with the patient’s medical history, his knowledge and expertise, ability to provide medical reports, and the awareness to avoid malpractice. In many studies, the physician’s professional knowledge has played a significant role in patient choice [27, 28]. In another study, although proximity and availability were more important in determining initial choice, in long-term care, the doctor’s professional proficiency was found to be more significant [13, 28]. Another study using the discrete choice experiment method found that the two main determinants of patient choice were physical examinations and a friendly attitude [9].

In our study, these characteristics were gathered under the heading of Patient-Doctor relationship and were listed in a similar way. On the other hand, various studies have emphasized the importance of other characteristics. When articles synthesizing qualitative research concerning Patient-Doctor relationship are analysed, it has been seen that many traits such as Patient-Doctor relationship, professional proficiency, awareness of the patients’ medical history, lifestyle and habits, communication skills, ethics, trust, defensiveness etc. have been allocated different values in different studies [2]. Another study of out-patients has shown that although the main preference is based on the doctor’s professional proficiency and the quality of care, the patient-doctor relationship comes fourth in the ratings [8].

The reason for these variations may be methodological differences. This may be due to the fact that many studies have classified similar concepts under different categories and therefore the categorical tendencies may have differentiated. We could have obtained different categories if we had grouped some of the sub-headings in our study differently. For example, we could have combined the sub-headings “s/he should know our family and children” and “the doctor should know me” under the “Patient-Doctor Relationship” column. In addition, the “I should have only one doctor”, and “if s/he changes, then everything changes” could have come under the “The Setting” column; and all could have been grouped under the heading of “Continuity in Care”.

Since this terminology was decided upon within the context of the focus group interviews, it was deemed more appropriate for them to be evaluated under the suitable headings.

On the other hand, in another phase of on going research which has not gone to print yet and in which 496 participants took part, all the factors obtained in the focus group interviews were not categorized, and nevertheless, factor analysis indicated a similar grouping preference [23].

It won’t be wrong to consider the fact that another explanation of the difference among studies may be due to factors such as age, illness, and faith etc. which may result in subjective preferences. As we mentioned earlier, it is for this reason that we implemented the AHP, believing it would reflect what is best for the individual rather than whether the choices are correct or incorrect. For example, in some studies such as in the discrete choice experiment model, the comparison within variables is important and may be determined accordingly. [19, 27]. A structural equality model may be used to pinpoint the most important factor. [10].

Once the criteria were prioritised accordingly, the model used in this study enabled us to analyse the main concept of the consistency of preferences made by the patients. Thus, it differs from other studies analysing weighting. Various statistical analyses and models have assumed that patients’ choices reflect their actual preference at a given point in time. According to the model applied in our study, the result indicates that 94.8 % of participants’ preferences are inconsistent. This situation indicates that the patients’ values, expectations and priorities may not have been reflected in their choices. Therefore, it appears that the results obtained with the AHP may be more realistic in the reflection of existing preferences. Furthermore, the main purpose of this study is to provide a model enabling patients to make a more consistent decision. Nevertheless, we didn’t use steps 2 of the AHP as it is known that these steps are concerned with establishing an instrument enabling the participants to change their choices until a consistent preference is reached. We didn’t implement these steps, as our main objective was to evaluate preference consistency. On the other hand, the weighting distribution among the factors of “doctor attributes” is unknown. There is a clear need to develop an instrument which will enable patients to evaluate choices such as patient compatibility, patient-doctor satisfaction, and also to do further research to clarify and expand this relationship. After the characteristics of the physicians became clear, this model can be used to predict patients’ physician selection in real world. This study determined priority of criteria which are considered in choosing a family physician irrespective of alternatives. These criteria also highlight the features to which a physician should pay attention.

## Conclusions

Selection criteria for choosing a FP were put in a priority order by using AHP model. Kategorilerin öncelik sıralaması analizinde Aile Hekimi’nin mesleki bilgi ve becerilerini içeren profesyonel özellikleri en önemli kriter olarak belirlenmiştir. Diğer kriterler ise öncelik sırasına göre sırasıyla; aile hekiminin hasta hekim ilişkisi, kişisel özellikleri, etik özellikleri ve sağlık hizmetinin verildiği ortam olarak belirlenmiştir.

These criteria can be used as measures for selecting alternative FPs in further researches.

## Abbreviations

AHP: Analytic hierarchy process
FP: Family physicians
CR: Consistency ratio
W: Priority vector
